# The effect of computerized decision support systems on cardiovascular risk factors: a systematic review and meta-analysis

**DOI:** 10.1186/s12911-019-0824-x

**Published:** 2019-06-10

**Authors:** T. Katrien J. Groenhof, Folkert W. Asselbergs, Rolf H. H. Groenwold, Diederick E. Grobbee, Frank L. J. Visseren, Michiel L. Bots, on behalf of the UCC-SMART study group

**Affiliations:** 1 Julius Center for Health Sciences and Primary Care, University Medical Center Utrecht, University of Utrecht, Heidelberglaan 100, 3584 CX Utrecht, the Netherlands; 2 Department of Cardiology, Division Heart & Lungs, University Medical Center Utrecht, University of Utrecht, Utrecht, The Netherlands; 3 Institute of Cardiovascular Science, Faculty of Population Health Sciences, University College London, London, UK; 4 Health Data Research UK and Institute of Health Informatics, University College London, London, UK; 5 Farr Institute of Health Informatics Research and Institute of Health Informatics, University College London, London, UK; 6 Department of Epidemiology, Leiden University Medical Center, Leiden, The Netherlands; 7 Department of Vascular Medicine, University Medical Center Utrecht, University of Utrecht, Utrecht, The Netherlands

**Keywords:** CDSS, Computerized decision support, Cardiovascular risk management

## Abstract

**Background:**

Cardiovascular risk management (CVRM) is notoriously difficult because of multi-morbidity and the different phenotypes and severities of cardiovascular disease. Computerized decision support systems (CDSS) enable the clinician to integrate the latest scientific evidence and patient information into tailored strategies. The effect on cardiovascular risk factor management is yet to be confirmed.

**Methods:**

We performed a systematic review and meta-analysis evaluating the effects of CDSS on CVRM, defined as the change in absolute values and attainment of treatment goals of systolic blood pressure (SBP), low density lipoprotein cholesterol (LDL-c) and HbA1c. Also, CDSS characteristics related to more effective CVRM were identified. Eligible articles were methodologically appraised using the Cochrane risk of bias tool. We calculated mean differences, relative risks, and if appropriate (I^2^ < 70%), pooled the results using a random-effects model.

**Results:**

Of the 14,335 studies identified, 22 were included. Four studies reported on SBP, 3 on LDL-c, 10 on CVRM in patients with type II diabetes and 5 on guideline adherence. The CDSSs varied considerably in technical performance and content. Heterogeneity of results was such that quantitative pooling was often not appropriate. Among CVRM patients, the results tended towards a beneficial effect of CDSS, but only LDL-c target attainment in diabetes patients reached statistical significance. Prompting, integration into the electronical health record, patient empowerment, and medication support were related to more effective CVRM.

**Conclusion:**

We did not find a clear clinical benefit from CDSS in cardiovascular risk factor levels and target attainment. Some features of CDSS seem more promising than others. However, the variability in CDSS characteristics and heterogeneity of the results – emphasizing the immaturity of this research area - limit stronger conclusions. Clinical relevance of CDSS in CVRM might additionally be sought in the improvement of shared decision making and patient empowerment.

**Electronic supplementary material:**

The online version of this article (10.1186/s12911-019-0824-x) contains supplementary material, which is available to authorized users.

## Background

The fast paced nature of medical science and practice challenge physicians to keep practicing concurrent to guidelines and the latest evidence. Some state that health care decision making has never been more complex because of multi morbidity and different severities of disease clustered in one individual [1]. Cardiovascular risk management (CVRM) in high-risk patients asks for a comprehensive approach and a lifelong effort of patients that affects lifestyle and dictates adherence to medical treatment of risk factors. CVRM is complex, because it involves a large number of risk factors that may change over time. CVRM guidelines provide support and advocate the use of risk prediction algorithms for the identification of patients at risk for (recurrent) cardiovascular events [2]. Treatment decisions, such as starting or intensifying lipid blood pressure lowering treatment are based on estimated absolute cardiovascular risk in individual patients and their absolute levels of risk factors. Yet, there seems to be a gap between guideline recommendations and daily clinical practice [3]. Adherence to guidelines varies between medical disciplines and between treating physicians, even for similar patients [3]. Completeness of risk factor assessment, pharmacological and non-pharmacological treatment initiation and long term uptake of treatment in patients with a cardiovascular condition can be further optimized, which potentially leads to reduction in to preventable cardiovascular morbidity and mortality [4, 5].

Computerized decision support systems (CDSS) are digital information systems that typically show a summary of patient data in an app, on a webpage, or within the electronic health record (EHR). In CVRM, CDSS can be used for reminders for assessment of risk factor levels, comprehensive presentation and evaluation of risk factor levels and cardiovascular risk estimates and for recommendation of evidence-based treatment modalities. This way, patient data and scientific evidence are incorporated into tailored strategies in daily practice [6]. CDSS have the potential to improve shared decision making, treatment adherence and eventually health outcome, without additional utilization of health-care resources [7].

Multiple apps and decision support systems have been and are being developed: over the last years, at least 16 systematic and 2 narrative reviews on the effectiveness of CDSS on practitioner performance and patient outcomes have been published [8–25]. But due to the large variation in CDSS functionalities – drug alerts, laboratory test ordering, treatment advice – clinical applications, and patient populations these reviews are restricted in terms of generalizability and applicability. Cardiovascular disease prevention is a multidisciplinary process in a population characterized by multimorbidity and multiple diseases of different severities. Preferably, a CDSS focused on CVRM should fit all these patients. Recently, Njie et al. reported a systematic review on CDSS in cardiovascular risk management, focusing on improvement in guideline adherence by physicians [26]. Complete assessment of vascular risk factors and guideline-adherent clinical testing increased) [26]. It would be even more clinically relevant to know whether use of CDSS would improve cardiovascular risk factor levels and ultimately reduce cardiovascular event rates. This is yet to be investigated.

This systematic review of randomized clinical trials evaluates the effects of CDSS on CVRM and aims to identify CDSS characteristics that are related to effective CVRM.

## Methods

This review was performed in accordance with the Preferred Reporting Items for Systematic Reviews (PRISMA) guidelines [27].

### Outcomes of interest

The primary focus of the systematic review was on absolute difference in and target attainment of blood pressure, LDL-c and HbA1c. The secondary focus was on the evidence based medicine practice performance of the user, defined as actions compliant to the guidelines applied in the specific studies. Lastly we investigated whether the technical embedding, the measurements provided, the level of evidence provided, the level of therapy advice provided and prompting were related to the likelihood of having positive, beneficial results.

### Study eligibility

Randomized controlled trials (RCT’s) and cluster RCT’s using CDSS as an intervention on CVRM were included. As parameters for CVRM the established, objectively measurable and pharmacological treatable risk factors were used: elevated blood pressure, impaired glycemic control and dyslipidemia. Although relevant in CVRM, effects on lifestyle factors such as smoking and physical inactivity were outside the scope of this review.

Studies were included if the CDSS was used for a patient specific advice given to the physician or about to make a treatment decision for the individual patient (not the group-effect). Studies on medical training or primary users other than physicians were excluded. Assessments of diagnostic or prognostic CDSS tools compared with routine care were excluded. Advice on prescription of medicine supported by CDSS was included, but drug prescribing error alarm systems were not. Clinical support using out-of-date systems (fax, paper flowcharts etc.) or trials focusing on implementation of an EHR were excluded.

### Search strategy

A systematic literature search was conducted in PubMed, Embase and Cochrane Library for publications up to March 20th 2018. A combination synonyms of “*computerized decision support systems*” AND (“*system performance*” OR “*hypertension”* OR “*dyslipidemia”* OR “*diabetes*”) AND “*randomized controlled trial*” and synonyms was used. The detailed search strategy is in Additional file 1.

### Data collection and assessment of methodological quality

A data collection form was designed prior to screening of the retrieved articles. KG subtracted the data from the retrieved articles. Uncertainties were resolved by discussion with an objective expert (Prof. Dr. R. Scholten). Additional quality assessment was performed using the Cochrane Collaboration’s tool to assess the risk of bias [28]. Blinding of participants and physicians was not possible due to the nature of the intervention. Therefore we excluded this criterion from the appraisal. All studies were scored on the randomization process, blinding of outcome assessment, attrition bias, reporting bias and other potential sources of bias. Additionally, cluster RCT’S were assessed on recruitment bias, baseline imbalance, loss of clusters, adjustments for clustering in the analysis and comparability with individually randomized trials. Items were scored low risk of bias, unknown risk of bias or high risk of bias. Studies with more than two items at ‘a high risk of bias’ were excluded from further analyses.

Data processing and additional analyses were performed in RevMan [29]. For continuous outcomes, we calculated the mean differences (MDs) with 95% confidence intervals (CI’s) between CDSS and usual care at follow up. For dichotomous outcomes, we calculated the relative risks with 95% CI’s between CDSS and usual care at follow up. If standard deviations were missing, we calculated them using reported CIs, standard errors, t and/or *p* values, according to the principles provided in the Cochrane handbook [30]. We assessed heterogeneity using the I^2^ [31]. Results were considered too heterogeneous for reliable pooling if the I^2^ was > 70% [32]. Where appropriate, results were pooled using a random-effects model.

## Results

**Fig. 1:**
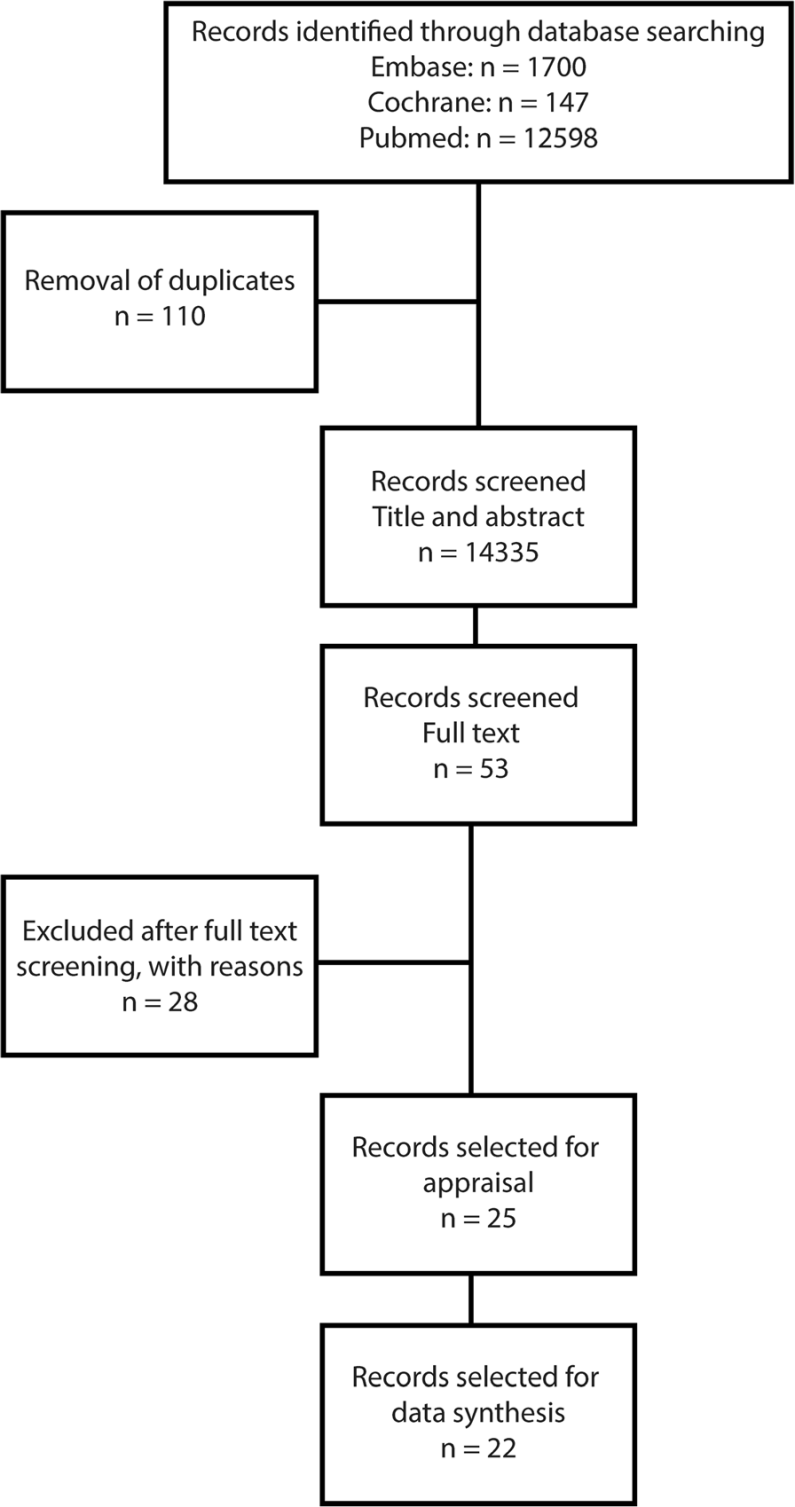
Flowchart

The systematic literature search (Additional file 1) yielded 14,335 unique articles. Figure 1 shows the number of articles that were retrieved from the searches, were reviewed and were included in the analysis. After selection based on title and abstract, 53 articles were considered potentially eligible for answering the research question. Full text screening followed. Three studies were excluded because of the study design: one study performed a retrospective cross sectional study [33], two studies performed a before and after study [34, 35]. Dorr et al. did not focus solely on CVRM interventions [36] and was excluded. Four studies featured interventions focusing on influencing patient behaviour [37], learning strategies [38], data search queries [39] or prediction models [40] that were non-compatible with our intervention of choice, and were excluded. Three articles focused on patient and physician satisfaction and/or CDSS uptake rather than measurable clinical outcomes and were excluded [41–43]. Ten studies did not answer our research question [44–52]. Three studies featured an out-of-date system, three focused on the implementation of an EHR [53–59]. Lipton et al. investigated glucose control at the intensive care unit, which was considered a too different population [60]. Also a study on a continuous monitoring system was excluded from further assessments [61]. That left us with 25 studies that were critically appraised.

### Critical appraisal

Randomization process was not sufficiently reported by two studies [62, 63] (Additional file 2. Critical appraisal table). Two studies reported considerable (up to > 20%) loss to follow up [62, 64]. Furthermore insufficient information was provided on the prevention of (selection and detection) bias by Saenz et al. [63] That left us with 17 articles on risk factor changes (Table 1) proceeded to data analysis and 5 articles providing evidence on guideline adherence.

**Table 1:** Characteristics of randomized trials on CDSS effect on BP or LDL cholesterol

Author, year of publication	Superiority/ Non-inferiority	Inclusion period	Follow up	Guideline adhered	Country	Type of practice	Prevention: primary, secondary or both	Number of participants		Age (mean (sd) or median (range)	
								CDSS	Usual Care	CDSS	Usual Care
Patients with an indication for CVRM											
Anchala et.al, 2015 [65]	Superiority	Aug 2011- March 2012	12 months	NR	India	PCP	Primary	840	783	NR ***	NR
Hicks et al, 2008 [66]	Superiority	July 2003 – Feb 2005	12 months	JNC VI + VII	USA	PCP	Both (*)	786	1048	64	61
Montgomery et.al, 2000 [67]	Superiority	Sept 1996 – Sept 1998	12 months	NR	UK	PCP	Both (1–17% secondary)	229	157	71 (6)	71 (5)
Roumie et. al, 2006 [68]	Superiority	July 2003 – Dec 2003	6 months	JNC-VII	USA	Hospital/PCP	Both (*)	547	324	65.5 (12.0)	65.1 (11.9)
Eaton et.al, 2011 [69]	Superiority	Oct 2004 - May 3005	12 months	ATP III	USA	PCP	Both (*)	2000	2105	46.7 ()6.3)	46.4 (8.4)
Gill et.al, 2009 [70]	NR	Nov 2005 – Oct 2006	12 months	ATP III	USA	PCP	Both (**)	26,696	37,454	NR ***	NR
Lester et al, 2006 [71]	Superiority	July 2003 – July 2004	12 months	NR	USA	PCP	Secondary	118	117	64.3 (14.5)	62.4 (13.3)
Patients with type II diabetes											
Ali et.al, 2016 [72]	Superiority	Jan 2011 – June 2012	24–36 months	ADA	India and Pakistan	Outpatient clinics	Both (6.8–39.4% secondary	575	571	54.2 (9.2)	54.2 (9.2)
Cleveringa et.al, 2008 [73]	Non- inferiority	March 2005 – Aug 2007	12 months	Dutch CVRM	NL	PCP	Both (47.1 and 63.3% secondary)	1699	1692	65.2 (11.3)	65.0 (11.0)
Glasgow et.al, 2005 [74]	Superiority	2001–2002	12 months	NR	USA	PCP	Both (*)	379	354	62 (1.4)	64 (1.3)
Grant et.al, 2008 [75]	Superiority	July 2005 – Sept 2007	12 months	NR	USA	PCP	NR	126	118	58.8 (10.1)	53.3 (12.3)
Holbrook et.al, 2009 [76]	Superiority	2002–2003	5.9 months (mean)	American/ Canadian Diabetes Association	Canada	PCP	Both (5.5–19% secondary)	253	258	61.0 (13.1)	60.5 (11.9)
Ilag et.al, 2003 [77]	Superiority	Oct 1999 – Sept 2000	2 years	NR	USA	University affiliated PCP	NR	83	71	59 (14)	59 (120
Maclean et.al, 2009 [78]	Superiority	June 2003 – Jan 2005	2 years	NR	Canada	PCP with hospital based clinical laboratories	NR	3886	3526	62.4 (19–99)	63.5 (18–97)
Mathers et.al, 2012 [79]	Superiority	2008–2011	12 moths	NICE	UK	PCP	Both (3.2–31.1% secondary)	95	80	66 (39–82)	62 (42–87
Meigs et.al, 2003 [80]	Superiority	May 1998 – April 1999	12 months	NR	USA	Hospital based internal medicine clinic	Both (52.4–50.9% secondary)	307	291	68 (12)	67 (12)
O’Connor et.al, 2011 [81]	NR	Oct 2006 – May 2007	6 months	NR	USA	PCP	Both (11.5–23.4% secondary)	1194	1362	57.0 (10.7)	57.5 (10.1)

* distributions not reported; ** only specified categories of cardiovascular risk (from total study population: 18.6% high risk; 16.4 medium risk, 65% low risk); *** Presented percentages per age category, no overall mean/median age

*NR* not reported, *RCT* randomized controlled trial, *PCP* primary care practice, *USA* United States of America, *UK* United Kingdom, *NL* The Netherlands, *JNC* Joint National Committee on Prevention, Detection, Evaluation and Treatment of High Blood Pressure, *ATP III* Adult Treatment Panel III, *ADA* American Diabetes Association, *NICE* the National Institute for health and Care Excellence. * Superiority although not powered for within site variation

### CVRM on elevated blood pressure

A total of 4 studies investigated blood pressure and blood pressure target attainment in CVRM patients (Table 1), of which 3 looked at mean blood pressure (Fig. 2.1) [65, 67, 68] and 3 at target attainment (Fig. 2.2) [65, 66, 68] . The studies were performed in primary care practices, mostly with a follow up of 12 months. The CDSS was integrated in the electronical health record in 3 studies [65–67]. All CDSS provided a risk factor overview, only 2 a cardiovascular risk score [65, 67]. Prompting occurred in 3 studies [65, 66, 68].

In the studies on absolute change in mean blood pressure, 1594 patients received CDSS care and 1237 usual care. The mean blood pressure decreased more in those with CDSS care compared to usual care in 2 out of 3 studies. The overall pooled mean difference in systolic pressure was − 1.49 mmHg (95%CI -5.861; 2.63), but heterogeneity of results (I^2^ = 67%) is only just below the maximum I^2^ for a reliable meta-analysis [65, 66, 68].

**Fig. 2:**
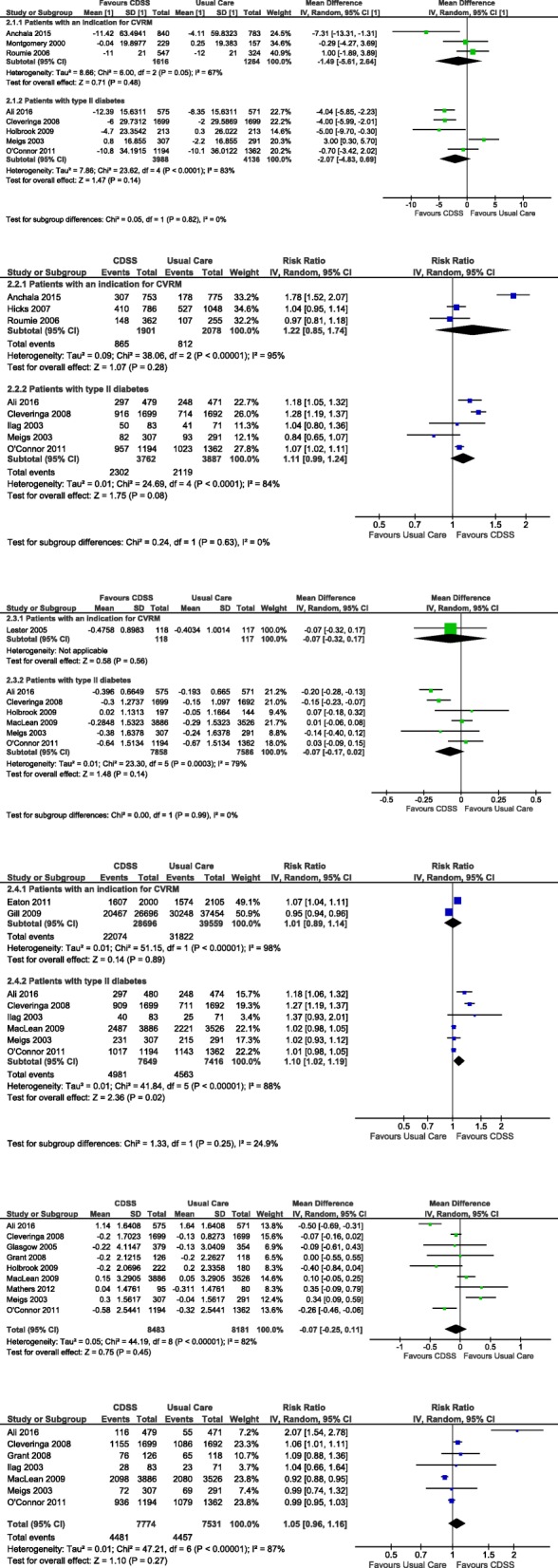
**2.1** Absolute change of mean systolic blood pressure (mmHg) at follow up in CDSS and usual care. **2.2** Prevalence of blood pressure target attainment at follow up in CDSS and usual care. **2.3** Absolute change of LDL-c change (mmol/L) at follow up in CDSS and usual care. **2.4** Prevalence of LDL-c target attainment at follow up in CDSS and usual care. **2.5** Absolute change in mean HbA1c in patients with type II diabetes (mmol/mol). **2.6** Prevalence of HbA1c target attainment in patients with type II diabetes at follow up in CDSS and usual care

In the studies on target attainment, 1901 patients received CDSS care and 2078 usual care. The number of patients reaching their blood pressure goal was higher with CDSS care than with usual care (pooled risk ratio [RR] 1.22 (95%CI 0.85; 1.74), but the heterogeneity of results (I^2^ = 95%) was too large to perform a reliable meta-analysis [65, 67, 68].

A total of 7 studies investigated blood pressure and blood pressure target attainment in patients with type II diabetes (Table 1), of which 6 studies investigated mean blood pressure [72, 73, 76, 80, 81] and 5 investigated target attainment [72, 73, 77, 80, 81]. The studies were performed in primary care practices, mostly with a follow up was 12 months. The CDSS was integrated in the electronical health record in 4 studies. All CDSS provided a risk factor overview, none a cardiovascular risk score. Prompting occurred in 1 study [72].

The overall pooled mean difference in systolic pressure and difference in number of patients reaching their blood pressure goal was comparable to the CVRM population: studies were relatively more positive towards CDSS care, but the heterogeneity of results (I^2^ > 83%) was too large to perform a reliable meta-analysis.

### CVRM on dyslipidemia

A total of 3 studies investigated lipid control in CVRM patients (Table 1), of which 1 study investigated mean LDL-c [71] and 2 investigated LDL-c target attainment [69, 70]. The studies were conducted in primary care practices with a follow up of 12 months [69–71].The CDSS was built within the electronical health record in two studies [70, 71]. Eaton et al. used a PDA based system and integrated patient support into the program [69]. All CDSS provided a risk factor overview, target support and medication support. None of the CDSSs’ featured a cardiovascular risk score. Prompting occurred in two studies [70, 71].

Only one study reported on change in mean LDL-c (slightly lower after CDSS (− 0.48 (0.08) mmol/L) than after usual care (− 0.41 (0.09) mmol/L; Fig. 2.3) [71]. The studies on target attainment showed contradictory results. On average, there was no difference between CDSS and usual care groups (RR 1.01 (95%CI 0.89; 1.14), I^2^ 98%) [69, 70].

A total of 6 studies investigated lipid control in patients with type II diabetes (Table 1), of which 5 studies investigated mean LDL-c [73, 76, 78, 80, 81] and 5 investigated LDL-c target attainment [72, 73, 77, 78, 80, 81].

The 6 studies targeting cholesterol performed in patients with type II diabetes (7858 CDSS, 7586 usual care) showed widely variable results for mean LDL-c change (I^2^ 79%, Fig. 2.3.1). A borderline statistically significant improvement of attained LDL targets was found in CDSS care compared to usual care (pooled RR 1.10(95% CI 1.02; 1.09), I^2^ 88%; Fig. 2.4.1).

### CVRM on glycemic control in diabetics

A total of 10 studies investigated glycemic control in patients with type II diabetes (Table 1), of which 9 studies investigated mean HbA1c [72–76, 78–81] and 7 investigated HbA1c target attainment [72, 73, 75, 77, 78, 80, 81]. Cleveringa et al. was the only study with a non-inferiority design [73]. Most studies were conducted in primary care practices and the follow up ranged from 6 months to 2 years. As far as described, three studies were EHR based [75, 77, 80], two web-applications [72, 76], one personal digital assistant (PDA)/tablet [79], 1 CD-rom [74] and one system using the EHR to extract data but using fax for promoting [78]. All studies provided a risk factor overview but none a cardiovascular risk score. Furthermore, two included patient motivating techniques into the intervention [75, 76]. Additionally, four studies organized a guideline instruction session [72, 73, 79, 81].

In the studies on absolute change in mean HbA1c, 8483 patients received CDSS care and 8181 usual care. The mean HbA1c seems to decrease more after CDSS care than after usual care (MD -0.07% (95% CI -0.25;-0.11%), I^2^ 82%; Fig. 2.5) [72–76, 78–81]. Similar prevalence HbA1c target attainment (CDSS *n* = 7774, usual care *n* = 7531) was found in care supported by CDSS and usual care (RR 1.05 (95%CI 0.96; 1.16); Fig. 2.6) [72, 73, 75, 77, 78, 80, 81]. There was insufficient homogeneity to reliably conduct pooled analysis (I^2^ 87%).

### Usual care characteristics

Detailed description of usual care was lacking in 20 of the 24 reports. Roumie et al. sent all participants an e-mail message that explained the planned intervention and provided physician education in all groups [68]. Similarly, a poster with a CVRM flowchart including which risk factors to assess, classification of risk instructions and advice for lifestyle interventions was provided to the usual care group in another study [65]. A patient activation tool was provided via a PDA with smoking cessation, weight loss, healthy diets, exercise and lipid lowering medication adherence materials and web access to calculate the heart-age in the study of Eaton et al. [69]. Grant et al. provided their controls with a questionnaire on family history and health maintenance journals [75].

### CDSS characteristics

Apart from the technical performance of CDSS, design and usability are important drivers behind the success of the systems. The interaction design can influence error through the length and proximity of selection items, bullet points and similar item descriptions [82]. A study on design characteristics showed that information should be displayed all at once and at one glance [83].

The CDSS characteristics of the studies included in this analysis are listed in Table 2. The cardiovascular risk score was only provided by two of the studies on blood pressure care, not in patients with type II diabetes [65, 67]. Advice on specific medication (name and dose) was not unanimously associated with improvement of the health outcomes [65, 66, 69, 70, 71, 75, 77, 79, 80]. Prompting of measurements and treatment strategies were associated with positive CDSS results in five out of seven studies [66, 68, 70–72, 78, 84]. Studies using prompting that were not associated with positive results used a web-link (not EHR) based [68] and fax reminders [78]. Almost all studies in patients with type II diabetes include patient activation in the CDSS intervention. The lack of insight into the automated computation and the source of information decreased user satisfaction [85, 86]. CDSS built in within the EHR were superior to usual care in seven out of nine studies. Non-EHR based systems CDSS were superior to usual care in three out of eight studies.

**Table 2:** CDSS characteristics and summary of results

	CDSS characteristics							Outcomes			
Author	Risk factor summary	Risk calculator/ score	Target support	Medication support	Prompting/reminders	Other	Technical basis	Clinical outcome			
								On target	Favours CDSS	Mean	Favours CDSS
Patients with an indication for CVRM											
Anchala et. al, 2015 [65]	+	+	+	+	+	CDSS training	EHR			BP	+
Hicks et al., 2008 [66]	+	–	+	+	+		EHR	BP	+/−		
Montgomery et. al, 2000 [67]	+	+	–	–	–	Training of nurse practitioners	EHR			BP	+/−
Roumie et.al, 2006 [68]	+	–	+	–	+	Weblink to JNC-7. All PCP’s received education	Computer sign-on alert	BP	+/−	BP	+/−
Eaton et.al, 2011 [69]	+	–	+	+	–	Computer kiosk with patient activating software	PDA	LDL-c	+		
Gill et.al, 2009 [70]	+	–	+	+	+		EHR	LDL-c	+/−		+/−
Lester et al, 2006 [71]	+	–	+	+	+		EHR			LDL-c	+/−
Patients with type II diabetes						Patients with type II diabetes					
Ali et.al, 2016 [19, 72]	+	–	–	–	+	Support by non-physician care coordinators. Patient focused. Web		HbA1c LDL-c BP	+ + +	HbA1c LDL-c BP	+ + +
Cleveringa et.al, 2008 [73]	+	–	+	+	–	Feedback every 3 months on target attainment to physician and patient	NR	HbA1c BP LDL-c	+ + +	HbA1c	+/− + +
Glasgow et.al, 2005 [74]	+	–	–	–	–	Development of self-management action plan	CD-ROM			HbA1c	+/−
Grant et.al, 2008 [75]	+	–	+	+	–	Questions enabling patient empowerment Patient focused	EHR	HbA1c	+/−	HbA1c	+/−
Holbrook et.al, 2009 [76]	+	–	+	–	–	Patient focused	Web			HbA1c	+/−
Ilag et.al, 2003 [77]	+	–	+/−	+	–	Reviewed by a nurse, advice emailed to PCP and entered into EHR	EHR	HbA1c	+/−	HbA1c BP LDL-c	+/− +/− +/−
Maclean et.al, 2009 [78]	+	–	–	–	+ (by fax)		Fax	HbA1c LDL-c	- +/−	HbA1c LDL-c	+/− +/−
Mathers et.al, 2012 [79]	+	–	–	+	–	PDA training. Probabilities of outcome. Patient value clarification	PDA			HbA1c	+/−
Meigs et.al, 2003 [80]	+	–	+	+	–	Single screen view	EHR	HbA1c BP LDL-c	+/− - +/−	HbA1c BP LDL-c	- +/− +/−
O’connor et.al, 2011 [81]	+	–	+	+	–	Training of nurses and physician. Reimbursement ($500–800)	EHR	HbA1c BP LDL-c	+/− + +/−	HbA1c BP LDL-c	+ + +/−

*EHR* electronical health record, *PCP* primary care practice, *PDA* personal digital assistant, *BP* blood pressure, *LDL-c* low-density lipoprotein cholesterol, *HbA1c* glycated hemoglobin, *JNC* Joint National Committee on Prevention, Detection, Evaluation and Treatment of High Blood Pressure

### Guideline adherence

Five RCT’s reported on general improvement of adherence to guidelines in patients with an indication for CVRM, with and without diabetes and/or a history of a cardiovascular event (Table 3) [87–91].

**Table 3:** Study characteristics for studies on guideline adherence

Author	Superiority/ Non-inferiority	Inclusion period	Follow up	Guideline adhered	Country	Type of practice
Goud et.al, 2009 [87]	Superiority	Jan 2005 - Dec 2005	12 months	Cardiac rehabilitation	The Netherlands	Rehabilitation center
Holbrook et.al, 2011 [88]	NR	April 2003–June 2005	12 months	NR	Canada	PCP
Mazzaglia et.al, 2016 [89]	Superiority	NR	12 months	ESC	Italy	PCP
Schnipper et.al, 2010 [90]	NR	March 2007 - Aug 2007	30 days	NR	USA	PCP
Sequist et.al, 2005 [91]	NR	Oct 2002 – April 2003	6 months	ADA/AHA	USA	PCP

*NR* not reported, *RCT* randomized controlled trial, *PCP* primary care practice, *USA* United States of America, *L* low risk of bias, *U* unknown risk of bias, *H* high risk of bias, *ESC* European Society of Cardiology, *AHA* American Heart Association, *ADA* American Diabetes Association

The CARDSS (cardiac rehabilitation decision support system) was implemented in 21 centers (including 2878 patients), focusing on exercise, education, relaxation and lifestyle change after a cardiac event. CDSS increased concordance with guideline recommended interventions by 3.5% (95%CI 0.1; 5.2%). Furthermore, CDSS significantly reduced both overtreatment (25.7% (95%CI 4.3; 54.1%)) and under treatment (42.8% (95%CI 1.1; 68.0%)) [87].

The COMPETE III (Computerization of Medical Practices for the Enhancement of Therapeutic Effectiveness) study assessed electronic vascular risk decision support in patients with increased risk or a history of a cardiovascular disease. The CDSS improved a composite process outcome with 4.70 points on a 27 point scale ((95%CI 3.63; 5.71) *p* < 0.001). The CDSS group reported a higher improvement of the continuum of care (odds ratio (OR) 4.18 (95%CI 3.04; 5.76), *p* < 0.001) and their ability to improve their health (OR 3.07 (95%CI 2.37; 3.99) *p* < 0.01) [88]. In patients with diabetes without and with a history of acute myocardial infarction (*n* = 3956) and stroke (*n* = 2158), more antiplatelet drugs (+ 2.7% vs + 0.15%; *p* < 0.001) and lipid lowering drugs (+ 4.2% vs. + 2.8%, *p* = 0.001) were prescribed in the CDSS group [89]. Another study amongst patients with diabetes (*n* = 4549) and coronary artery disease (CAD, *n* = 2199) also showed an improvement of diabetes care (OR 1.30 95%CI 1.01; 1.67) and coronary heart disease risk management (OR 1.25 (95%CI 1.01, 1.55)) amongst the CDSS patients [91]. Lastly, in a study in > 7000 patients in primary care showed an increase in the number of deficiencies addressed amongst patients with diabetes or CAD (CDSS 11.4% vs. normal care 10.1% (OR 1.14 (95%CI 1.02, 1.28), *p* = 0.01) [90].

## Discussion

A systematic review and meta-analysis was conducted to provide insight into the effects of computerized decision support systems (CDSS) on cardiovascular risk factor levels and identify characteristics of CDSS related to improved care. A considerable number of CDSS for CVRM were developed, but a clear clinical benefit is absent. Some features of CDSS seem more promising than others. However, the variability in approaches of CDSS and heterogeneity of the results limit stronger conclusions.

Due to differences in technical basis, content and comparison group, comparability of the studies included in this review is limited. CDSS vary greatly in technical approaches, as well as to how and to which extent they support the physician. In addition, or as a result, there was large heterogeneity in the results of the studies, such that definite conclusions cannot be drawn from the pooling of findings. Moreover, the usual care group was ill defined such that it was impossible to understand what care was delivered as standard. Visual inspection of funnel plots investigating the relationship between effect and sample size does not show a distinct publication bias (Additional file 3). But it is still possible that positive results on CDSS are more likely to get published. These aspects limit strong recommendations on success factors in use of CDSS. Also, our analyses were restricted by what was reported: some studies reported raw means, prevalence or odds ratios, others reported adjusted and/ or imputed effect measures [72, 76, 78, 84]. We were unable to incorporate these differences into our analyses. An easy, but major step forward would be to apply the existing guidelines for reporting trial results using drug or devices in the publications and registration in national or international trial registries.

Although very relevant in CVRM, effects on lifestyle factors such as smoking and physical inactivity were outside the scope of this review. CDSS focus on guideline adherent management measured by change in pharmacological treatment and risk factor profiles without registering meta-information on the decisions: how did the CDSS affect counselling by the physician and the shared decision making process? Helping the physician remember to address smoking can be achieved by CDSS, but hów to address this has proven to be an important factor in the uptake [92]. Lifestyle interventions in particular need a more personal approach that is directed towards coaching and long-term engagement, rather than incidental support of guideline adherent management (the goal of CDSS). Current literature is very positive about such smoking cessation interventions: there is consistent evidence that web-based and mHealth smoking cessation interventions may increase abstinence moderately [92].

Though the results are conflicting and strength of conclusions is limited, the findings from this study can perhaps be used to give direction to future developments. More evidence is needed on long-term evaluations including assessment of the effect on treatment adherence and vascular event rate [93]. This could the also include other relevant cardiovascular risks such as anti-coagulation therapy for patients with atrial fibrillation to prevent stroke, cardio protective medicine in heart failure patients to prevent death or cardiac function replacement therapy, and fluid balance in patients witch chronic kidney disease to prevent renal replacement therapy. The paucity of good quality studies, with sufficient sample size and follow up, on clinical outcomes hinders interpretation and restricts transposing these results into clinical practice. This again emphasizes that guidance for generating relevant evidence needs to be followed and taken up as requirement for funding and publication of novel developments [94]. In designing a study investigating the effect of CDSS, the CDSS’s life cycle should be taken into account including the type of device, the intended use and users, and working mechanism. The device should be investigated in its natural habitat: applied to the same target individuals and in the same setting as in usual care. The complex interplay between device performance, user skills and learning curves has to be incorporated methodologically [95]. The regulatory environment, including guidance on scientific evaluation, regulations and legislation and privacy issues needs to evolve together with this emerging field of health technology.

CDSS in principle enable the physician to integrate evidence and patient information into tailored strategies for daily practice and increase guideline adherence [6, 26]. In this review, it seems that the technical basis, prompting and the type of information provided influence the effect of the CDSS on cardiovascular risk factor improvement. Also, patient involvement in the CDSS process seems to increase the effects. Roumie et al. added patient education in a third study arm: 59.5% of the patients in this group were on target regarding their blood pressure (compared to usual care: RR 1.31 (95%CI 1.06; 1.62)). This significant improvement could be driven by increased patient empowerment. Patient empowerment can be defined as a personal disposition (patient’s control over medical strategies) and as a relational concept (collaborative patient-doctor relation) and has been shown to significantly increase the compliance to therapy [96–98]. The Lancet Commission of Hypertension also suggests patient empowerment as one of the strategies to address the global burden of hypertension in the future [99].

## Conclusion

In conclusion, we did not find a clear clinical benefit from CDSS in cardiovascular risk factor levels and target attainment. Some features of CDSS seem more promising than others. However, the variability in CDSS characteristics and heterogeneity of the results – emphasizing the immaturity of this research area - limit stronger conclusions. Clinical relevance of CDSS in CVRM might additionally be sought in the improvement of shared decision making and patient empowerment.

## Additional files

Additional file 1: Systematic search strategy. (DOCX 14 kb)

Additional file 2: Critical appraisal table. (DOCX 22 kb)

Additional file 3: Funnel plots. (DOCX 41 kb)

## Data Availability

Not applicable, all data is extracted from published articles.

## Abbreviations

CAD: Coronary artery disease
CDSS: Computerized decision support system
CI: Confidence interval
CVRM: Cardiovascular risk management
EHR: Electronic health record
HbA1c: Glycated hemoglobin
LDL-c: Low-density lipoprotein cholesterol
MD: Mean difference
OR: Odds ratio
PDA: Personal device assistant
RCT: Randomized controlled trial
RR: Risk ratio
SBP: Systolic blood pressure
