# Supplementary material for: The effect of computerized decision support systems on cardiovascular risk factors: a systematic review and meta-analysis
Source: BMC Med Inform Decis Mak. 2019 Jun 10;19:108. doi: 10.1186/s12911-019-0824-x (PMC6558725; doi:10.1186/s12911-019-0824-x)
Supplement: Supplementary file 1 — Systematic search strategy. (DOCX 14 kb) [file 12911_2019_824_MOESM1_ESM.docx]

**Supplement 1. Search strategy**

PubMed

(((((((((((((((((((((((((((((((Decision Support Systems, Clinical[MeSH Terms]) OR Decision Support Systems, Clinical[Title/Abstract]) OR Clinical Decision Support Systems[Title/Abstract]) OR Clinical Decision Support[Title/Abstract]) OR Clinical Decision Supports[Title/Abstract]) OR Decision Supports, Clinical[Title/Abstract]) OR Support, Clinical Decision[Title/Abstract]) OR Supports, Clinical Decision[Title/Abstract]) OR Decision Support, Clinical[Title/Abstract]) OR Decision Support Systems, Management [MeSH Terms]) OR Decision Support Systems [Title/Abstract]) OR Medical Order Entry Systems[MeSH Terms]) OR Medical Order Entry Systems[Title/Abstract]) OR Order Entry Systems, Medical[Title/Abstract]) OR Medication Alert Systems[Title/Abstract]) OR Alert System, Medication[Title/Abstract]) OR Medication Alert System[Title/Abstract]) OR System, Medication Alert[Title/Abstract]) OR Alert Systems, Medication[Title/Abstract]) OR Computerized Physician Order Entry System[Title/Abstract]) OR Computerized Provider Order Entry System[Title/Abstract]) OR CPOE[Title/Abstract]) OR Computerized Provider Order Entry[Title/Abstract]) OR Computerized Physician Order Entry[Title/Abstract]) OR Clinical Decision-Making[MeSH Terms]) OR Clinical Decision-Making[Title/Abstract]) OR Clinical Decision Making[Title/Abstract]) OR Decision-Making, Clinical[Title/Abstract]) OR Medical Decision-Making[Title/Abstract]) OR Decision-Making, Medical[Title/Abstract]) OR Medical Decision Making[Title/Abstract]))

AND

( Technology Assessment, Biomedical[MeSH Terms]) OR Technology Assessment, Biomedical[Title/Abstract]) OR Assessment, Technology[Title/Abstract]) OR technology assessment[Title/Abstract]) OR Health Technology Assessment[Title/Abstract]) OR Health Technology Assessments[Title/Abstract]) OR Technology Assessment, Health[Title/Abstract]) OR Assessment, Health Technology[Title/Abstract]) OR Assessments, Health Technology[Title/Abstract]) OR Technology Assessments, Health[Title/Abstract]) OR Assessment, Biomedical Technology[Title/Abstract]) OR Assessments, Biomedical Technology[Title/Abstract]) OR Biomedical Technology Assessments[Title/Abstract]) OR Technology Assessments, Biomedical[Title/Abstract]) OR Assessments, Technology[Title/Abstract]) OR technological characteristics[Title/Abstract]) OR informatics[MeSH Terms]))

OR

(visualization[Title/Abstract]) OR design[Title/Abstract]) OR infographics[Title/Abstract]) OR display format[Title/Abstract]) OR presentation format[Title/Abstract]) OR format[Title/Abstract] OR feedback[Title/Abstract])

OR

(((((reimbursement[Title/Abstract]) OR guidelines[Title/Abstract]) OR legislation[Title/Abstract]) OR guideline[Title/Abstract]) OR law[Title/Abstract]) OR legal[Title/Abstract]

OR

(((((((((((((((((((((((Dyslipidemias[MeSH Terms]) OR Dyslipidemias[Title/Abstract]) OR Hypercholesterolemia[MeSH Terms]) OR Hypercholesterolemia[Title/Abstract]) OR Hyperlipoproteinemias[MeSH Terms]) OR Hyperlipoproteinemias[Title/Abstract]) OR Hyperlipemia[MeSH Terms]) OR Hyperlipemia[Title/Abstract]) OR Dyslipidemia[Title/Abstract]) OR Dyslipoproteinemias[Title/Abstract]) OR Dyslipoproteinemia[Title/Abstract]) OR High Cholesterol Levels[Title/Abstract]) OR High Cholesterol[Title/Abstract]) OR High Cholesterol level[Title/Abstract]) OR cholesterol, high[Title/Abstract]) OR Hypercholesteremia[Title/Abstract]) OR elevated cholesterol[Title/Abstract]) OR cholesterol, elevated[Title/Abstract]) OR HDL[Title/Abstract]) OR high-density lipoprotein[Title/Abstract]) OR LDL[Title/Abstract]) OR low-density lipoprotein[Title/Abstract]) OR cholesterol[Title/Abstract]) OR triglycerids[Title/Abstract])

OR

((((Hypertension[MeSH Terms]) OR Hypertension[Title/Abstract]) OR Hypertensive[Title/Abstract]) OR high blood pressure[Title/Abstract])

OR

(((((diabetes mellitus[MeSH Terms]) OR diabetes mellitus[Title/Abstract]) OR DM[Title/Abstract]) OR diabetes[Title/Abstract]) OR T2DM[Title/Abstract]) OR T1DM[Title/Abstract])

Cochrane

Decision Support Systems, Clinical:ti,ab OR Clinical Decision Support Systems:ti,ab OR Clinical Decision Support:ti,ab OR Clinical Decision Supports:ti,ab OR Decision Supports, Clinical:ti,ab OR Support, Clinical Decision:ti,ab OR Supports, Clinical Decision:ti,ab OR Decision Support, Clinical:ti,ab OR Decision Support Systems :ti,ab OR Medical Order Entry Systems:ti,ab OR Order Entry Systems, Medical:ti,ab OR Medication Alert Systems:ti,ab OR Alert System, Medication:ti,ab OR Medication Alert System:ti,ab OR System, Medication Alert:ti,ab OR Alert Systems, Medication:ti,ab OR Computerized Physician Order Entry System:ti,ab OR Computerized Provider Order Entry System:ti,ab OR CPOE:ti,ab OR Computerized Provider Order Entry:ti,ab OR Computerized Physician Order Entry:ti,ab OR Clinical Decision-Making:ti,ab OR Clinical Decision Making:ti,ab OR Decision-Making, Clinical:ti,ab OR Medical Decision-Making:ti,ab OR Decision-Making, Medical:ti,ab OR Medical Decision Making:ti,ab

AND

(Technology Assessment, Biomedical:ti,ab OR Assessment, Technology:ti,ab OR technology assessment:ti,ab OR Health Technology Assessment:ti,ab OR Health Technology Assessments:ti,ab OR Technology Assessment, Health:ti,ab OR Assessment, Health Technology:ti,ab OR Assessments, Health Technology:ti,ab OR Technology Assessments, Health:ti,ab OR Assessment, Biomedical Technology:ti,ab OR Assessments, Biomedical Technology:ti,ab OR Biomedical Technology Assessments:ti,ab OR Technology Assessments, Biomedical:ti,ab OR Assessments, Technology:ti,ab OR technological characteristics:ti,ab

OR

visualization:ti,ab OR design:ti,ab OR infographics:ti,ab OR display format:ti,ab OR presentation format:ti,ab OR format:ti,ab OR feedback:ti,ab

OR

reimbursement:ti,ab OR guidelines:ti,ab OR legislation:ti,ab OR guideline:ti,ab OR law:ti,ab OR legal:ti,ab

OR

Dyslipidemias:ti,ab OR Hypercholesterolemia:ti,ab OR Hyperlipoproteinemias:ti,ab OR Hyperlipemia:ti,ab OR Dyslipidemia:ti,ab OR Dyslipoproteinemias:ti,ab OR Dyslipoproteinemia:ti,ab OR High Cholesterol Levels:ti,ab OR High Cholesterol:ti,ab OR High Cholesterol level:ti,ab OR cholesterol, high:ti,ab OR Hypercholesteremia:ti,ab OR elevated cholesterol:ti,ab OR cholesterol, elevated:ti,ab OR HDL:ti,ab OR high-density lipoprotein:ti,ab OR LDL:ti,ab OR low-density lipoprotein:ti,ab OR cholesterol:ti,ab OR triglycerids:ti,ab

OR

Hypertension:ti,ab OR Hypertensive:ti,ab OR high blood pressure:ti,ab

OR

diabetes mellitus:ti,ab OR DM:ti,ab OR diabetes:ti,ab OR T2DM:ti,ab OR T1DM:ti,ab)

Embase

decision support system/exp OR ‘decision making’:ab,ti , computer-assisted; decision support; decision support systems, management; decision support techniques

AND

(‘hypertension’/exp OR‘hypertension’:ab,ti OR ‘high blood pressure’:ab,ti OR ‘systolic pressure’:ab,ti OR ‘diastolic pressure’:ab,ti OR ‘blood pressure’:ab,ti

OR

‘hypercholesterolemia’/exp OR ‘hypercholesterolemia’:ab,ti OR ‘hypercholesterolaemia’:ab,ti OR ‘cholesteremia’:ab,ti OR ‘cholesterinemia’:ab,ti OR ‘cholesterolemia’:ab,ti OR ‘hypercholesteremia’:ab,ti OR ‘hypercholesterinaemia’:ab,ti OR ‘hypercholesterinemia’:ab,ti OR ‘hypercholesterolaemia’:ab,ti OR ‘hypertryglyceridemia’:ab,ti OR ‘hypertriglyceridaemia’:ab,ti OR ‘high density lipoprotein’/exp OR ‘high density lipoprotein’:ab,ti OR ‘high density lipoprotein cholesterol’/exp OR ‘high density lipoprotein cholesterol’:ab,ti OR ‘HDL’:ab,ti OR ‘HDL cholesterol’:ab,ti OR ‘cholesterol’:ab,ti OR ‘low density lipoprotein’/exp OR ‘low density lipoprotein’:ab,ti OR ‘low density lipoprotein cholesterol’/exp OR

‘low density lipoprotein cholesterol’:ab,ti OR ‘LDL’:ab,ti OR ‘LDL cholesterol’:ab,ti OR ‘dyslipidemia’/exp OR ‘dyslipidemia’:ab,ti OR ‘dyslipidaemia’:ab,ti OR ‘Lipid’:ab,ti

OR

‘diabetes mellitus’/exp OR ‘diabetes’:ab,ti OR ‘diabetic’:ab,ti OR ‘insulin insensitivity’/exp OR ‘insulin insensitivity’:ab,ti OR ‘insulin resistance’:ab,ti OR ‘Insulin resistance syndrome’:ab,ti OR ‘glucose intolerance’/exp OR ‘glucose intolerance’:ab,ti )
