# Supplementary material for: The effect of computerized decision support systems on cardiovascular risk factors: a systematic review and meta-analysis
Source: BMC Med Inform Decis Mak. 2019 Jun 10;19:108. doi: 10.1186/s12911-019-0824-x (PMC6558725; doi:10.1186/s12911-019-0824-x)
Supplement: Supplementary file 2 — Critical appraisal table. (DOCX 22 kb) [file 12911_2019_824_MOESM2_ESM.docx]

**Supplement 2. Critical appraisal table**

| Author, year | Randomization  unit | Randomization process | Allocation concealment | | Blinding of outcome assessment | Attrition bias (incomplete outcome assessment) | Reporting bias (selective reporting) | | Other potential sources of bias. | Recruitment bias | Baseline imbalance | Loss of clusters | Analysis adjusted for cluster correlation | Comparable with individually randomized trials | |
| --- | --- | --- | --- | --- | --- | --- | --- | --- | --- | --- | --- | --- | --- | --- | --- |
| Patients with an indication for CVRM | | | | | | | | | | | | | | |  |
| Anchala, 2015 | PCP (cluster) | Low | Low | | High | Low | Low | | Low | Low | Low | Low | Yes | Yes | |
| Bertoni, 2009 | PCP (cluster) | Low | Unknown | | Unknown | High | Low | | Low | High | Low | High | Yes | Yes | |
| Hicks, 2007 | Individual | Unknown | Unknown | | Low | Low | Low | | Low | - | - | - | - | - | |
| Montgomery, 2000 | PCP (cluster) | Low | Low | | Unknown | Unknown | Low | | Low | Low | Low | Low | Yes | Yes | |
| Roumie, 2006 | Individual | Low | Low | | Unknown | Low | Unknown | | Low | Low | Low | High | Yes | Yes | |
| Eaton, 2011 | PCP (cluster) | Low | Low | | Low | Low | Low | | Low | Low | Low | Low | Yes | Yes | |
| Gill, 2009 | Individual | Low | Unknown | | Low | Low | Unknown | | Low | - | - | - | - | - | |
| Lester, 2005 | Individual | Low | Low | | Low | Low | Unknown | | Unknown | - | - | - | - | - | |
| Murray, 2004 | Individual | Unknown | Unknown | | High | High | Unknown | | Low | - | - | - | - | - | |
| Patients with type II Diabetes | | | |  | | |  |  | |  |  |  |  |  | |
| Ali, 2016 | Individual | Low | Low | | Low | Low | Low | | - | - | - | - | - | - | |
| Cleveringa, 2008 | PCP (cluster) | Low | Low | | Unknown | Low | Low | | Low | Low | Low | Low | Yes | No | |
| Glasgow, 2005 | PCP (cluster) | Low | Low | | Low | High | Unknown | | Low | Low | Low | Low | Yes | Yes | |
| Grant, 2008 | PCP (cluster) | Low | Unknown | | Low | High | Low | | Low | Low | Low | Low | Yes* | No | |
| Holbrook, 2009 | Individual | Low | Unknown | | Low | Low | Low | | Low | - | - | - | - | - | |
| Ilag, 2003 | PCP (cluster) | Low | Unknown | | Unknown | Low | High | | Low | Low | Low | Low | Yes | Yes | |
| Maclean, 2009 | PCP (cluster) | Low | Low | | Low | Low | Low | | Low | Low | Low | Low | Yes | No | |
| Mathers, 2012 | PCP (cluster) | Low | Low | | Unknown | Low | Low | | Low | Low | Low | Low | Yes | No | |
| Meigs, 2003 | Clinician (cluster) | Low | Unknown | | Low | Low | Unknown | | Low | Low | Low | Low | Yes | Yes | |
| O’Connor, 2011 | Individual | Low | Low | | Unknown | Low | Low | | Low | - | - | - | - | - | |
| Saenz, 2012 | PCP (cluster) | High | Unknown | | High | Unknown | High | | Low | Low | Low | Unknown | No | Yes | |
| Studies on guideline adherence | | | | | | | | | | | |  |  |  | |
| Goud, 2009 | Rehabilitation Centers (cluster) | Low | Low | | Low | Low | Low | | Low | Low | Low | High | Yes | Yes | |
| Holbrook, 2011 | Individual | Low | Unknown | | Low | Low | Low | | Low | - | - | - | - | - | |
| Mazzaglia, 2016 | Clinician (cluster) | Low | Low | | Low | Low | Unknown | | Low | Low | Low | Low | No | Yes | |
| Schnipper, 2010 | Clinician (cluster) | Low | High | | Low | Low | Low | | Low | Low | Low | Low | Yes | Yes | |
| Sequist, 2005 | PCP (cluster) | Unknown | Low | | Low | Low | Unknown | | Low | Low | High | Low | Yes | Yes | |

* this was unclear after reading the manuscript, but was checked via correspondence with the first author.

Legend: CVRM – cardiovascular risk management; PCP – primary care practice.
