# Supplementary material for: The effect of computerized decision support systems on cardiovascular risk factors: a systematic review and meta-analysis
Source: BMC Med Inform Decis Mak. 2019 Jun 10;19:108. doi: 10.1186/s12911-019-0824-x (PMC6558725; doi:10.1186/s12911-019-0824-x)

**Supplement 4. Funnel plots**

Figure 8. Funnel plot of the studies reporting the mean blood pressure difference

**
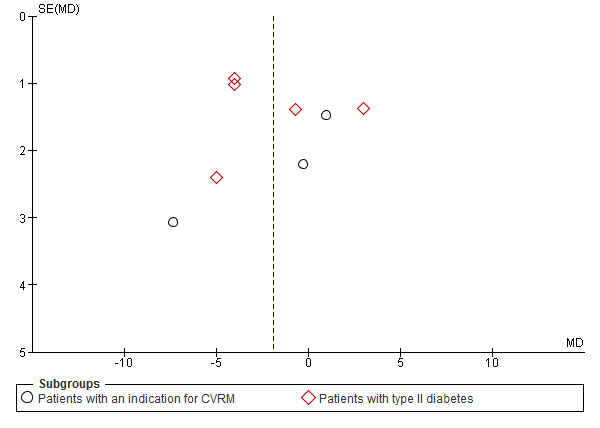
**

Figure 9. Funnel plot of the studies reporting the relative risk of blood pressure target attainment

**
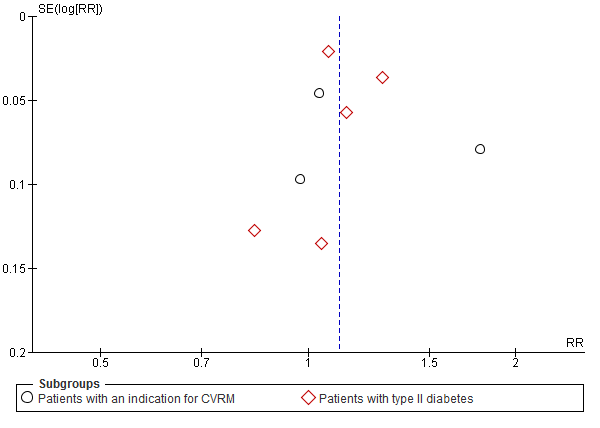
**

Figure 10. Funnel plot of the studies reporting the mean difference in LDL-c

**
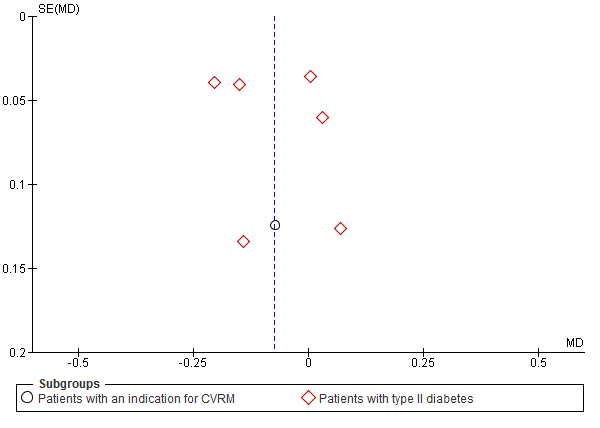
**

Figure 11. Funnel plot of the studies reporting the relative risk of LDL-c target attainment

**
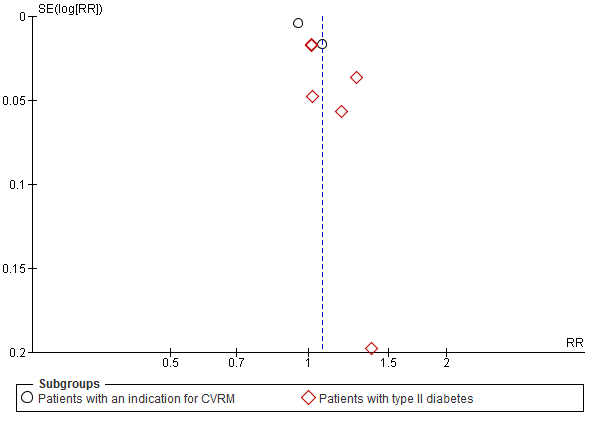
**

Figure 12. Funnel plot of the studies reporting the mean difference in HbA1c

**
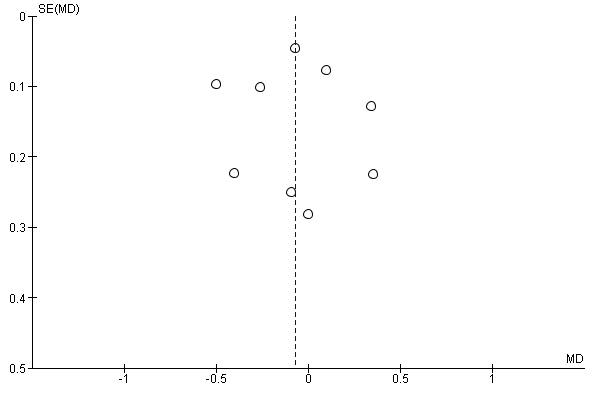
**

Figure 13. Funnel plot of the studies reporting the relative risk of HbA1c target attainment


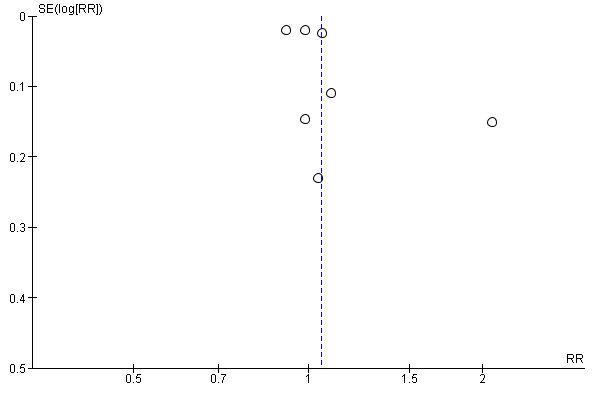

Supplement: Supplementary file 3 — Funnel plots. (DOCX 41 kb) [file 12911_2019_824_MOESM3_ESM.docx]
